# A High-Performance Coniform Helmholtz Resonator-Based Triboelectric Nanogenerator for Acoustic Energy Harvesting

**DOI:** 10.3390/nano11123431

**Published:** 2021-12-17

**Authors:** Haichao Yuan, Hongyong Yu, Xiangyu Liu, Hongfa Zhao, Yiping Zhang, Ziyue Xi, Qiqi Zhang, Ling Liu, Yejin Lin, Xinxiang Pan, Minyi Xu

**Affiliations:** 1 Dalian Key Lab of Marine Micro/Nano Energy and Self-Powered System, Marine Engineering College Dalian Maritime University, Dalian 116026, China; yuanhc@dlmu.edu.cn (H.Y.); yuhongyong2020@dlmu.edu.cn (H.Y.); simonlxy@dlmu.edu.cn (X.L.); zyp672216686@dlmu.edu.cn (Y.Z.); yyds@dlmu.edu.cn (Z.X.); qiqizhang@dlmu.edu.cn (Q.Z.); pinky@dlmu.edu.com (L.L.); 2 Tsinghua-Berkeley Shenzhen Institute, Tsinghua Shenzhen International Graduate School, Tsinghua University, Shenzhen 518055, China; zhaohf21@mails.tsinghua.edu.cn; 3 School of Electronics and Information Technology, Guangdong Ocean University, Zhanjiang 524088, China

**Keywords:** coniform Helmholtz resonator, triboelectric nanogenerator, acoustic energy harvesting

## Abstract

Harvesting acoustic energy in the environment and converting it into electricity can provide essential ideas for self-powering the widely distributed sensor devices in the age of the Internet of Things. In this study, we propose a low-cost, easily fabricated and high-performance coniform Helmholtz resonator-based Triboelectric Nanogenerator (CHR-TENG) with the purpose of acoustic energy harvesting. Output performances of the CHR-TENG with varied geometrical sizes were systematically investigated under different acoustic energy conditions. Remarkably, the CHR-TENG could achieve a 58.2% higher power density per unit of sound pressure of acoustic energy harvesting compared with the ever-reported best result. In addition, the reported CHR-TENG was demonstrated by charging a 1000 μF capacitor up to 3 V in 165 s, powering a sensor for continuous temperature and humidity monitoring and lighting up as many as five 0.5 W commercial LED bulbs for acoustic energy harvesting. With a collection features of high output performance, lightweight, wide frequency response band and environmental friendliness, the cleverly designed CHR-TENG represents a practicable acoustic energy harvesting approach for powering sensor devices in the age of the Internet of Things.

## 1. Introduction

In the era of the Internet of Things (IoT), it is highly desired for the development of sensor devices that are environmentally friendly, independent and operation maintenance-free [1,2]. Since various forms of energy exist in the working environment of sensor facilities, such as solar energy, vibration energy, wind energy, sound energy, etc., energy harvesting from the environment provides a practical solution for the sustainable power supply for sensor equipment [3,4,5,6,7]. Among different energy forms in environment, as a green, abundant and sustainable energy source, acoustic energy is gaining the intrigue of energy harvesting researchers [8,9,10,11]. However, acoustic energy is mainly wasted due to its low energy density of sound waves and the lack of practical energy harvesting technologies [12]. If such a widely distributed energy were collected, converted into electricity and utilized in a managed manner, it would provide an essential solution for the self-powering of the IoT sensor nodes. Nevertheless, most previous studies focused on piezoelectricity or electromagnetic induction have inevitable disadvantages, such as low electrical output performance or complex structure design [13,14,15,16,17]. Hence, it is imperative to develop acoustic energy harvesting devices with high electrical output performance and reasonable practicability.

In 2012, Professor Wang Zhonglin reported a new approach to transforming environment low-frequency energy into electrical energy by utilizing a Triboelectric Nanogenerator (TENG) [18]. With its features of low cost, light weight, easy manufacture and high power density, TENG demonstrates great competitiveness in low-frequency energy collection and has been deemed to be the most prospective approach to achieve distributed energy harvesting (vibration energy [19,20,21,22], wind energy [23,24,25,26], wave energy [27,28,29,30], and acoustic energy [31,32,33,34]) and self-powered sensing [35,36,37,38]. Among the studies on acoustic energy harvesting by utilizing TENG, Yang et al. [39] first explored the TENG acoustic energy harvester based on organic film, amazingly, by utilizing a Helmholtz resonator, the device achieves a maximum output voltage of 60.5 V, a maximum output current of 15.1 μA and a maximum power density of 60.2 mW/m^2^ output performance at the acoustic frequency of 240 Hz. Regarding innovation structure of the TENG acoustic energy harvester, Cui et al. [40] investigated a novel mesh TENG acoustic energy harvester that could operate in a wide frequency range (50~425 Hz). The mesh structure design enhances the electrical output performance of the apparatus, which can generate a current density of 45 mA/m^2^ with a maximum open-circuit voltage of 90 V. More recently, Wang et al. [41] reported a new sound-driven TENG with an integrated embroidery hoop, which could generate 500 V, 124 μA electricity output. Although the device is simple in design and fabrication, it cannot achieve the convergence and reinforcement of acoustic energy. In terms of structural innovation for enhanced acoustic energy collection, the Helmholtz resonator is of great help to strengthen the energy harvesting effect. Particularly, our group has designed an improved Helmholtz resonator-based TENG for efficient acoustic energy harvesting [33] in which dual tubes were installed on the outside of the resonator. Through optimized design, the sound pressure sensitivity of TENG per area reaches 1.23 V/Pa·cm^2^, and the power density per unit acoustic pressure reaches 1.82 W/Pa·cm^2^, which achieves the best sound energy collection and power generation effect in the field. However, there is very little research on the internal structure optimization of Helmholtz resonator-based TENG for high-performance acoustic energy collection. It is still essential and necessary to further improve the performance of acoustic energy harvesting and widen the frequency range of collected sound for developing self-powered devices based on acoustic energy.

Herein, we developed a novel coniform Helmholtz resonator Triboelectric Nanogenerator (CHR-TENG) for efficient collection of acoustic energy in environment. The CHR-TENG is composed of a coniform Helmholtz resonance cavity, an aluminum film with uniformly distributed acoustic holes and a fluorinated ethylene propylene (FEP) film with conductive ink printed electrodes. To identify the characteristics of acoustic energy harvesting effect, output performances of the CHR-TENG with different geometrical sizes under different acoustic conditions were systematically investigated. Compared with the conventional acoustoelectric conversion devices, the CHR-TENG demonstrates better output performance due to its capture and reinforcement effects on sound energy.

## 2. Results and Discussion

### 2.1. Structure Design of the CHR-TENG

**Figure 1:**
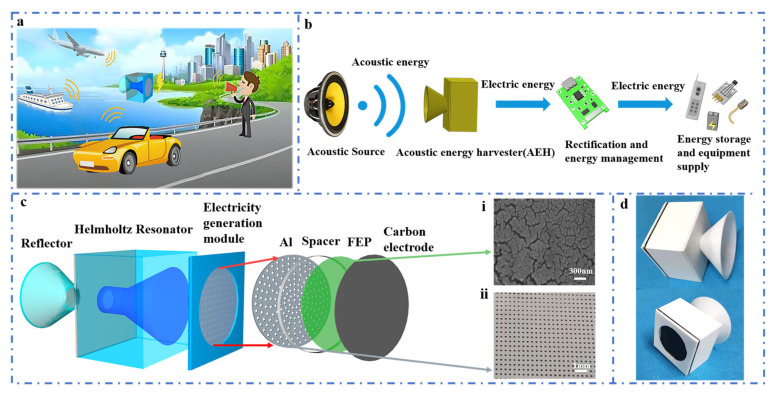
The structure design of the CHR-TENG. (**a**) Schematic diagram of various applications of the CHR-TENG in acoustic energy harvesting. (**b**) Schematic diagram of the acoustic energy harvesting and management system. (**c**) A schematic illustration of the CHR-TENG. (**d**) Physical images of the CHR-TENG.

Figure 1 depicts the structure design of the CHR-TENG, which can be used in various acoustic generation sources for energy harvesting for self-powering wireless sensor nodes, such as transportation scenarios of vehicles, airplanes or ships, where there have frequent activities and monitoring are needed. Generally speaking, acoustic levels in the transportation scenarios are mainly in the form of low frequency and high sound pressure level. Detailed acoustic levels can be seen Table S1 in Supplementary Materials. Figure 1b displays the schematic of the acoustic energy harvesting system: specifically, the acoustic source produces acoustic wave energy in the vibration form; the acoustic energy collection device focuses the energy and then utilizes the acoustic–electric conversion technology to convert acoustic waves from vibration energy to electrical energy; the electricity output is rectified by the energy management module with optimal impedance matching, which is ultimately used to power sensors, electrical energy storage or small power supplies. The detailed structure design of the CHR-TENG is illustrated in Figure 1c. The CHR-TENG consists of a front-end acoustic gathering structure (3D plastic printed, hereafter named as “reflector” for the sake of simplification), a coniform Helmholtz resonance cavity (3D plastic printed) and an electricity generation unit. The electricity generation unit comprises an aluminum membrane with uniformly distributed acoustic holes etched by laser (Figure 1c-i) and an FEP membrane with conductive ink-printed electrodes. A spacer cavity is designed and inserted between the FEP membrane and the aluminum membrane to realize a better contact separation effect. The conical Helmholtz resonance cavity is a kind of acoustic resonance system that amplifies the acoustic energy through the resonance cavity and enhances the output performance of the TENG by resonance effect. FEP film and aluminum are common materials for TENG energy harvesting devices. FEP material is highly electronegative in nature, resulting in a large amount of charge transfer when it separates from the aluminum electrode in contact. Surface treatment of FEP contributes to reinforce output performance of the TENG [42]. In this work, to improve the output performance of the CHR-TENG, FEP film was polished to increase its surface roughness with surface features scanned by electron microscopy (Figure 1c-ii). Figure 1d displays the physical images of the CHR-TENG.

### 2.2. Working Principle of the CHR-TENG

**Figure 2:**
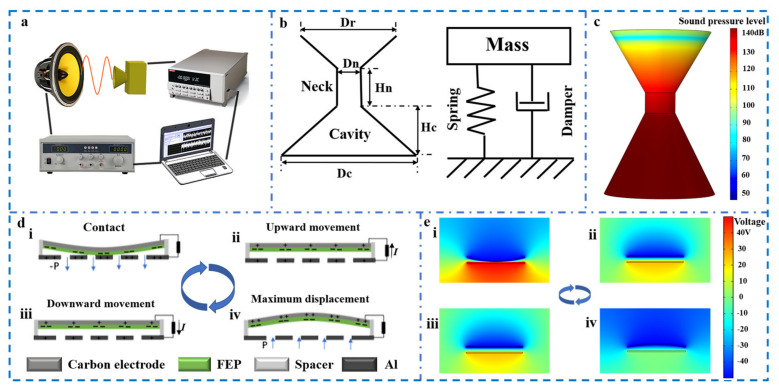
Working principle of the CHR-TENG. (**a**) Schematic diagram of acoustic energy collection experimental process. (**b**) The coniform Helmholtz resonator simplified by the mass–spring–damper system. (**c**) Distribution of sound pressure levels of the coniform Helmholtz resonator at the 1st resonant frequency. (**d**) The working mechanism of the CHR-TENG. (**e**) Periodic potential distribution change of the two electrodes simulated by COMSOL.

Figure 2 depicts the working principle of the CHR-TENG. Figure 2a illustrates the schematic diagram of the acoustic energy harvesting experimental process. The signal generator provides a stable and frequency adjustable sine wave electrical signal, and the power amplifier amplifies power of the electrical signal to drive the loudspeaker to generate acoustic waves. The acoustic frequency of the system can be adjusted by the signal generator, and the sound pressure level is controlled by the power amplifier. The FEP film in the CHR-TENG is driven by acoustic force to periodically separate from the aluminum electrode, generating a corresponding change in the electrical signal. The electrical output signals could be detected by an electrostatic high-resistance meter, received by a data acquisition card, and then plotted into a real-time change curve by LABVIEW software on the computer. The sound level meter is placed next to the FEP membrane, with measurement accuracy and resolution of 1.5 dB and 0.1 dB, respectively. To ensure accuracy and consistency of the experiments, the same TENG unit was used in all electricity generation performance and comparison experiments conducted in this study to exclude the systematic bias of the experiments. Since the resonator geometric dimension of the CHR-TENG is far less than that of the incident acoustic wavelength, the coniform Helmholtz resonator could be simplified as a 1D lumped system [43]. As schematically illustrated in Figure 2b, the coniform Helmholtz resonator is simplified to a mass–spring–damper model. Once driven by outside acoustic waves, the air inside the coniform Helmholtz cavity will operate as an air spring, while the air in the neck area will oscillate like a mass, forcing the air spring to expand and contract back and forth. Viscous losses generated due to damping mainly come from the friction of oscillating air at the neck region and radiation losses at the neck end. Formulation of the resonance frequency of the coniform cavity can be expressed as:

(1) $$F_{r}=\frac{C}{2\pi }\sqrt{\frac{{3A}_{n}}{{4V}_{c}H_{n}}}$$

where c is the speed of sound in air, A_n_ is the neck area, V_c_ represents the volume of the resonant cavity and H_n_ is the neck length. Figure 2c shows the distribution of sound pressure levels of the coniform Helmholtz resonator at the first resonant frequency. It can be seen from the simulation that the acoustic pressure is the highest at the bottom of the resonator cavity. A higher sound pressure in the cavity will result in a better resonance effect and, thus, a higher electrical output of the CHR-TENG. Therefore, the TENG electrical powering unit is placed at the bottom of the resonator cavity to ensure the best electrical output performance. Figure 2d presents a schematic depiction of the working mechanism of the CHR-TENG. The acoustic wave propagation causes the pressure between the FEP membrane and aluminum electrode to change periodically, generating repetitive vibrations of the FEP up and down. When the FEP membrane contacts the aluminum electrode, electron transfer is caused by electronegativity difference between the two materials, thus resulting in a negatively charged FEP surface and a positively charged aluminum electrode (Figure 2d-i). As the FEP film separates from the aluminum electrode caused by the acoustic pressure change, to equalize the local electric field, free electrons will flow from the conductive ink electrode to the aluminum electrode through external circuit, thus generating positive charges (Figure 2d-ii). Electron flow continues to the maximum distance between the two contact surfaces (Figure 2d-iii). After that the acoustic waves push the FEP film toward the aluminum electrode. During this stage, the voltage difference diminishes, and free electrons inside the aluminum electrode pass through an external circuit back to the conductive ink electrode (Figure 2d-iv). Ultimately, surfaces of the FEP film and the aluminum electrode come into contact again, and the charge distribution returns to the initial condition (Figure 2d-i). Hereto, the whole electricity generation cycle of the CHR-TENG completes, and the output of alternating current pulses under the action of acoustic waves is generated. To further investigate the acoustic field distribution of the conical Helmholtz resonator and the potential distribution change between electrodes of the CHR-TENG, COMSOL was employed to carry out finite element analysis, the simulation results of which are shown in Figure 2e,f, respectively. Simulation conditions are provided in the Supplementary Materials. The acoustic field distribution of the coniform resonator is illustrated in Figure 2e. The sound pressure level gradually increases from the opening to the end in the reflector structure, indicating a good sound convergence effect. To ensure the optimum output performance of the CHR-TENG, the TENG power generation unit is placed at the bottom of the coniform resonator, where the acoustic pressure level reaches the maximum. Figure 2f depicts the electrical potential distribution change between the FEP and the aluminum electrode.

### 2.3. Performance of the CHR-TENG

**Figure 3:**
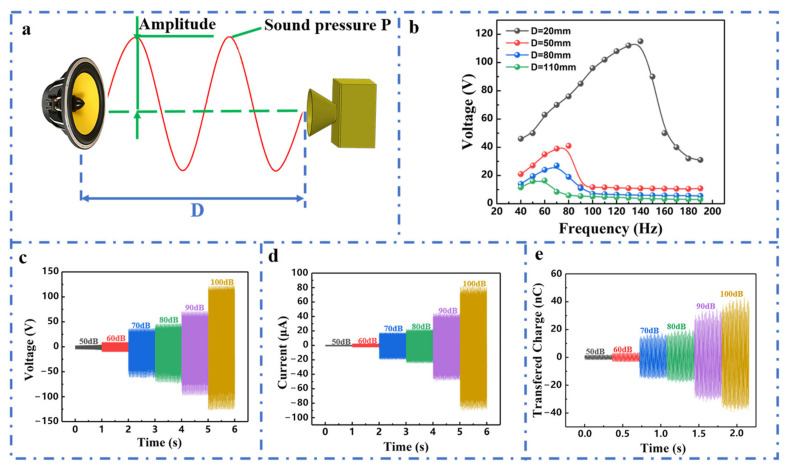
Electrical output performance of the CHR-TENG under different sound energy conditions. (**a**) Schematic diagram of acoustic wave propagation and capture. (**b**) Open-circuit voltage output of the CHR-TENG under the excitation of acoustic waves with different frequencies in distances ranging from 20 to 110 mm. (**c**) Open-circuit voltage, (**d**) short-circuit current and (**e**) transferred charge output of the CHR-TENG under acoustic excitation with pressure levels varying from 50 to 100 dB.

The electrical output performance of the CHR-TENG is influenced by the acoustic characteristics. This work systematically investigated the effects of acoustic pressure levels as well as distances to acoustic sources on the output performance of the CHR-TENG. Figure 3a depicts a schematic diagram of acoustic wave propagation and capture. The influence of the distance between the CHR-TENG and the sound source on the output performance was first investigated. Figure 3b shows output curves of the peak open-circuit voltage of the CHR-TENG at different distances in specific acoustic frequency (acoustic frequency range 40 to 200 Hz), with acoustic pressure levels ranging from 64 to 96.4 dB. Experimental results show that the output performance of the CHR-TENG gradually decreases with the increase of distance from the acoustic source. When the distance from the acoustic source was 20 mm, the CHR-TENG generated the peak open-circuit voltage 115 V at 140 Hz. When the distance increased from 20 mm to 110 mm, the CHR-TENG produced the peak open-circuit voltage of 18 V at 59 Hz. The output performance trend of short-circuit current and transferred charge is also consistent with that of the open-circuit voltage, which is due to the fact that output performance of the CHR-TENG is influenced by the coupling of acoustic propagation and electricity generation. In practical applications, we can choose the solution of getting as close to the acoustic source as possible or designing the array harvesters to enhance the energy harvesting and improve the output performance. The CHR-TENG operates in the pattern of contact and separation, whose control equation can be specified as:

(2) $$V_{\mathrm{oc}}=\frac{\sigma \cdot x(t)}{\varepsilon_{0}}$$

where V_oc_ is open-circuit voltage, σ is charge density, x(t) is film displacement and ε_0_ is dielectric constant. It can be seen from the formula that the electric output performance of the CHR-TENG is proportional to the film displacement when the material is selected constant. The effect of the sound pressure level and the distance from the acoustic source on the output performance of the CHR-TENG were investigated as the two elements that will influence the film displacement. In addition, the electricity output of the CHR-TENG was measured with acoustic pressure levels ranging from 50 dB to 100 dB. As shown in Figure 3c, the output voltage increases as the acoustic pressure increases. This is mainly attributed to the fact that the increase in acoustic pressure results in increased radial displacement of the FEP film. As the acoustic pressure levels increased from 50 dB to 100 dB, the open-circuit voltage increased from 4 V to 270 V, the short-circuit current rose from 0.1 to 85 μA (Figure 3d) and the transfer charge increased from 3 to 84 nC (Figure 3e).

**Figure 4:**
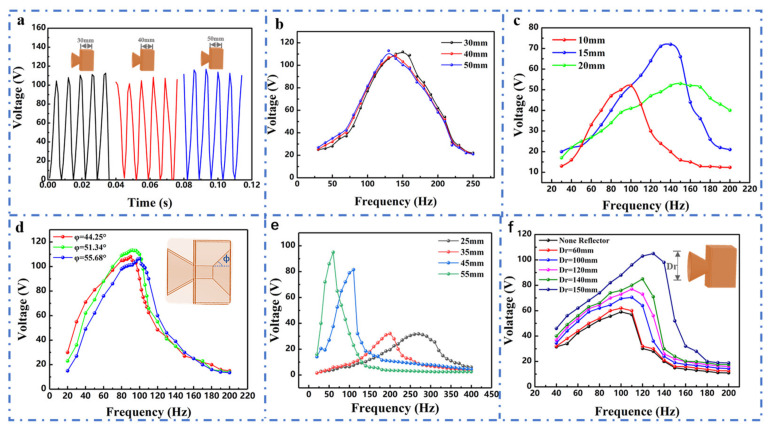
Electric output characterization of the CHR-TENG with varied geometrical sizes. (**a**) The maximum peak open-circuit voltage output of the CHR-TENG with different resonator thicknesses. (**b**) Open-circuit voltage output of the CHR-TENG with different resonator thicknesses. (**c**) Open-circuit voltage output of the CHR-TENG with different cross-sectional areas. (**d**) Open-circuit voltage output of the CHR-TENG with different coniform angles. (**e**) Open-circuit voltage output of the CHR-TENG with varied FEP film sizes. (**f**) Open-circuit voltage output of the CHR-TENG with different reflector sizes.

Among all the parameters in the resonance frequency formulation, V_c_ is one of the main influencing parameters on the resonance frequency of the resonator. The variation of the V_c_ will change the resonance frequency of the resonator and, thus, further influence the output performance of the CHR-TENG under the combined action of the electricity generation module. In order to explore the influence of V_c_ on the output performance of the CHR-TENG, we prefer to change the resonator thickness to change the resonator volume. To investigate the influence of the resonator’s thickness on electrical output performance of the CHR-TENG, we studied the output of the CHR-TENG with resonator thicknesses of 30 mm, 40 mm and 50 mm, respectively. As shown in Figure 4a, generally, the maximum peak open-circuit voltage outputs of the three CHR-TENGs have little difference: the maximum peak open-circuit voltage of the CHR-TENG with the thickness of 30 mm is 112 V, and the difference between 40 mm and 50 mm is not much, within only 5 V. Furthermore, a comparison is conducted to explore the open-circuit voltage outputs of the CHR-TENGs with different thicknesses at the same acoustic conditions (acoustic frequency 40–250 Hz, sound pressure level 60.3–92 dB).

As illustrated in Figure 4b, the acoustic frequency of generating the peak open-circuit voltage (optimal response frequency) decreased from 140 Hz to 130 Hz when the resonator’s thickness changed from 30 mm to 50 mm. This is due to the fact that the increase of the resonator thickness adds the resonator’s volume V_c_, and the resonator frequency will be lower with a bigger V_c_. However, it can be seen in the present experimental conditions that the enhancement of the acoustic wave by the resonator cavity does not improve as V_c_ changes. To investigate the influence of the cross-sectional area of the connecting tube on the electric output performance, three CHR-TENGs with different cross-sectional areas were employed to measure the output power under the acoustic condition of frequency range 30 to 200 Hz and sound pressure level range 60.2 to 83.5 dB, as the results are shown in Figure 4c. As the cross-sectional area increases, the power output varies in different frequency ranges. The optimal response frequency increases with the cross-sectional area; meanwhile, the acoustic response band is also broadened. However, the output performance of the CHR-TENG does not get better with increased area. This result is because the acoustic energy entering the resonator cavity is less when the cross-sectional area is small. However, when the cross-sectional area is too large, a good mass–spring damping system cannot be formed, resulting in poor acoustic pressure level implication. Therefore, the cross-sectional area of the CHR-TENG can be designed and selected based on the acoustic frequency band of the actual application scenarios. As the coniform angle is an important parameter of the coniform cavity structure, a comparison was conducted by varying coniform angles of the resonator cavity of the CHR-TENG, in which the experiment was carried out under the acoustic environment of frequency 20–200 Hz and sound pressure level 61–90.4 dB. The peak open-circuit voltages of the CHR-TENG were measured with coniform angles of 44.25, 51.34 and 55.68, respectively. It was found that the open-circuit voltage of the CHR-TENG would be an increasing and then decreasing trend for each one of the conical angles, as shown in Figure 4d. When the coniform angle was 44.25, the output voltage increased from 30 V to 108 V (the optimal output) as the frequency changed from 20 to 90 Hz, and then the output voltage gradually decreased to 15 V with the increasing sound frequency. The peak output voltage was 113.3 V for the CHR-TENG with a conical angle of 51.34, while the peak output voltage was 106.5 V for the CHR-TENG with a conical angle of 55.68. Obviously, the optimal response frequency of the CHR-TENG increases gradually with the increase of cone angle, but the increase is not large. The circular FEP film with fixed edges is driven by the air pressure difference generated by the acoustic source and vibrates reciprocally. The vibration of the circular FEP film is influenced by its size. We have investigated the influence of film size on the power output of the CHR-TENG.

As displayed in Figure 4e, the optimal response acoustic frequency changed from 270 Hz (FEP film diameter 25 mm) to 60 Hz (FEP film diameter 55 mm) with the increase of the FEP film size, while the optimal peak voltage for the optimal response acoustic frequency increased from 31.8 V to 96 V. More interestingly, the response bandwidth of the CHR-TENG with high output performance becomes narrower as the optimal response frequency decreases. The higher output results from the larger FEP film deformation, which can provide basic guidance for modulating the acoustic frequency response of the CHR-TENG. To enhance the acoustic energy collection, the front-end sound converging structure is installed on the CHR-TENG; hereafter, it is named as the reflector for the sake of simplification. The reflector on the CHR-TENG could effectively gather acoustic energy into the resonance cavity, thus contributing to improve its efficiency of acoustic–electric conversion. To systematically investigate the power output of the CHR-TENG for sufficient collecting acoustic energy into the resonance cavity, reflectors with diameters from 60 to 150 mm were employed to measure the output performance under the acoustic conditions of frequency from 40 to 200 Hz and sound pressure levels from 62.5 to 98.5 dB, as the results are shown in Figure 4f. Obviously, with the increase of reflector’s diameter (D_r_), the output peak open-circuit voltage of the CHR-TENG increases gradually. This is mainly ascribed to the fact that the increase in D_r_ results in a larger acoustic energy act on the CHR-TENG, thus, a better output performance. Meanwhile, the increase of the reflector’s diameter is equivalent to the increase of S in Equation (1). The optimal response frequency of the CHR-TENG increases with the reflector diameter, which shows that experimental results are consistent with the theoretical results. Therefore, to enhance the efficiency of acoustic–electric conversion, a sufficiently large reflector should be installed as far as the environmental space allows.

### 2.4. Demonstration of the CHR-TENG

**Figure 5:**
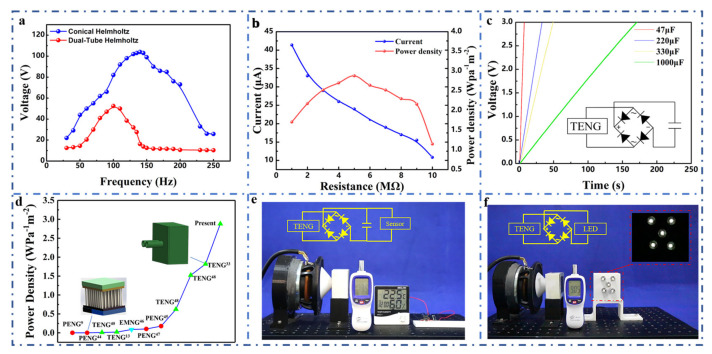
Demonstrations of the CHR-TENG for acoustic energy harvesting as a sustainable power source. (**a**) Comparison of the open-circuit voltage of the CHR-TENG and the dual-tube HR-TENG under excitation of the same acoustic conditions. (**b**) External resistance effect on the output performance of the CHR-TENG. (**c**) Charging of different capacitors by the CHR-TENG electrical output. (**d**) Power density comparison with previously acoustic energy harvesters. (**e**) Demonstration of the CHR-TENG for powering the sensor. (**f**) Demonstration of the CHR-TENG for powering LEDs.

To the best of our knowledge, the maximum power density per unit area structure in the published articles is the dual-tube Helmholtz resonated TENG (HR-TENG) proposed by our group [33]. Figure 5a shows the peak open-circuit voltage of the two acoustic energy harvesting TENGs under the excitation of the same acoustic waves. The open-circuit voltage of the CHR-TENG can achieve approximately 110 V at the optimal electrical performance output frequency of 140 Hz, which is 83.3% higher than the maximum open-circuit voltage of the dual-tube HR-TENG. In terms of the electrical output performance at various acoustic frequencies, electric output of the dual-tube HR-TENG under a frequency of 30 Hz to 50 Hz raised relatively slowly (from 12 V to 14 V); the output voltage increased from 14 V to 60 V when the frequency changed from 50 Hz to 100 Hz. After that, with the increase of the sound frequency, the peak voltage of the HR-TENG declined rapidly. When the frequency rose to 150 Hz, the open-circuit voltage output dropped to about 14 V; then, with the increase in frequency, the output voltage remained unchanged, which indicates that the response frequency of the dual-tube HR-TENG is between 50 and 150 Hz. As for the output performance of the CHR-TENG, the peak open-circuit voltage increased from 20 V to 110 V under the sound frequency of 30 Hz to 140 Hz, and then the output performance gradually decreased with the acoustic frequency increase. When the frequency increased to 240 Hz, the peak open-circuit voltage dropped to about 24 V and then remained unchanged with the increase of frequency, showing that the response frequency of the CHR-TENG is between 30 and 240 Hz. Throughout the 30 to 250 Hz sound frequency, the output voltage of the CHR-TENG is persistently above that of the dual-tube HR-TENG, indicating that the CHR-TENG has a better output performance and a wider response frequency.

To investigate the maximum output power of the CHR-TENG, the study was carried out by matching an external load to the acoustic energy harvesting device. Figure 5b depicts the effect of external resistance on the CHR-TENG output performance for the acoustic conditions of 140 Hz and 90 dB. As the external load resistance increases from 1MΩ to 10MΩ, the peak open-circuit current of the CHR-TENG gradually decreases, and the output power of the CHR-TENG for the same acoustic conditions first increases and then decreases (shown in Figure S7 in Supplementary Materials), showing a maximum power output when the load resistance is 5 MΩ. To make an equivalent comparison of output performance of various kinds of acoustic energy harvesters, the power density per unit of sound pressure is calculated. Particularly, the maximum power density of the CHR-TENG is 2.88 W/Pa·m^2^. Experimental results show that the output performance of the CHR-TENG improved considerably (a 58.2% higher power density) compared to the previously published work by utilizing the coniform Helmholtz resonator to enhance the acoustic energy harvesting. A more detailed comparison of power density with previous acoustic energy harvesters is shown in Table S2 in the Supplementary Materials. The coniform Helmholtz resonator can enhance the acoustic energy harvesting, and, thus, more acoustic energy is transformed to electricity through TENG. Both the resonator cavity volume and the coniform angles would affect the output performance under different acoustic conditions. Therefore, a high-performance CHR-TENG could be designed and selected through structure optimization and applied in a specific acoustic scenario.

Figure 5c presents a demonstration experiment of the CHR-TENG charging capacitors with various capacities. Alternating current output of the CHR-TENG is supplied to the capacitor ends through the rectifier bridge, and the electricity is then stored into the capacitor. Under the supply of the CHR-TENG, it took only about 6 s to charge a 47 μF capacitor to 3 V and about 165 s to charge a 1000 μF capacitor to 3 V, showing that the CHR-TENG has favorable charging performance for the capacitor. The power density of the CHR-TENG was calculated and compared with previously reported acoustic energy harvesters [9,13,32,33,44,45,46,47,48], as depicted in Figure 5d. Comparison of power density with previously acoustic energy harvesters is shown in Table S2 in Supplemental Materials. It can be seen from the figure that the optimal result in the literature has the power density per unit sound pressure 1.82 W/Pa·m^2^, while ours has that of 2.88 W/Pa·m^2^, showing a large improvement compared to the previous studies. In addition, as plotted in Figure 5e, the temperature and humidity sensor can be successfully switched on after charging the 1000 μF capacitor to 1.5 V. The sensor can function continuously while being powered by the CHR-TENG. Figure 5f shows the CHR-TENG can successfully light up as many as five 0.5 W commercial LED bulbs for acoustic energy harvesting by transforming acoustic energy to electricity. To examine the output performance of the acoustic energy harvester in real scenarios, the experimental results of the CHR-TENG testing in a ship’s engine room and the stability of the device was performed. The power output performance of the CHR-TENG in a ship’s engine room and stability test are shown in detail in Figures S6 and S8. Results show that the output capability of the harvester becomes weak due to the energy spreading in a wide space. In our future work, we will carry out further research by setting arrayed CHR-TENGs to eliminate the noise interference and adapting to the wideband acoustic frequency in a real application scenario. As for the stability test, experimental results indicate that there is hardly any change in the output performance after three days of tests, showing a good output stability characteristic.

## 3. Experimental Section

### 3.1. Fabrication of the CHR-TENG

The CHR-TENG consists of a coniform Helmholtz resonant cavity, an aluminum electrode with uniformly distributed acoustic holes and an FEP membrane with an ink-printed electrode. The front sound converging structure (or reflector) is fixed on the resonant cavity, and a coniform structure is designed to enhance the acoustic energy inside the resonator. Detailed dimensions of the CHR-TENG’s resonant cavity are D_r_ 60 mm, D_n_ 15 mm, H_n_ 10 mm, H_c_ 20 mm and D_c_ 45 mm, respectively. An aluminum electrode with uniformly distributed sound holes (diameter 0.2 mm) is utilized as an electropositive friction layer, whose radius and thickness are 22.5 mm and 0.1 mm, respectively. The flexible FEP film is used as the electronegative frictional electric material with the thickness of 30 μm, and the radius of the working area is 22.5 mm. As the FEP film is insulative, this work is designed to transfer electrons by attaching conductive ink electrodes with micron thickness on the backside of the FEP film. Resonator housings of various shapes and sizes are produced by a 3D printer utilizing PLA materials with high levels of device precision. The high filling density housing has flat surface and excellent airtightness, which benefits sound waves reflection and the formation of the resonance effect.

### 3.2. Characterization and Electrical Output Measurement of the CHR-TENG

When measuring the power output performance, the CHR-TENG is mounted on an optical board with a loudspeaker (JBL) producing sound, which is driven and adjusted by a function generator (YE1311) with sine waves. The sound is transmitted through a power amplifier (SA-5016) with an accuracy and resolution of 1.5 db and 0.1 db respectively. The output signals include open-circuit voltage, short-circuit current and transferred charge, which can be measured with a Keithley 6514 electrostatic meter.

### 3.3. Numerical Simulation

COMSOL Multiphysics was used for simulating the sound pressure distribution of the coniform Helmholtz resonator cavity. A classic acoustics model of a resonating circuit with a known theoretical solution is applied. The membrane is simplified to be a rigid one. The environment outside the cavity is regarded as the standard atmospheric pressure. The inlet sound pressure is set to be the value of pressure measured in front of the cavity. Based on the above assumptions, a pressure acoustics–frequency domain physics is set up in a two-dimensional axisymmetric model with the parameters and geometry of the CHR-TENG. After the integration is set, the models are solved by the Asymptotic Waveform Evaluation solver and the frequency domain–modal solver.

## 4. Conclusions

In summary, we proposed a low-cost, easily fabricated and high-performance coniform Helmholtz resonator-based TENG for efficient acoustic energy harvesting. In the CHR-TENG, the coniform Helmholtz resonator plays a role in enhancing the collection of acoustic energy. Driven by the harvested acoustic energy, the FEP membrane in the CHR-TENG alternately contacts and separates from the aluminum electrode, thus generating continuous electricity output. The novel design in the CHR-TENG could improve its output performance and broaden its response band over harvesting acoustic energy. Compared with the conventional acoustic energy harvesting TENG devices, the CHR-TENG has improved output performance. Output performances of the CHR-TENG with varied geometrical sizes were systematically investigated under different sound energy conditions, and results showed that, with the optimized design, the maximum acoustic sensitivity per unit area of the CHR-TENG could reach 1.68 V/Pa·m^2^, while the power density per unit of sound pressure was 2.88 W/Pa·m^2^. In addition, the CHR-TENG was demonstrated to charge a 1000 μF capacitor up to 3 V in 165 s, power a sensor for continuous temperature and humidity monitoring and light up as many as 464 commercial LED bulbs for acoustic energy harvesting. The newly designed CHR-TENG not only provides an effective guide for efficient conversion of acoustic energy into electrical energy but also demonstrates the potential application of the CHR-TENG in powering electronic devices.

## Data Availability

The data used to support the findings of this study are available from the corresponding author upon request.

## Supplementary Materials

The following are available online at https://www.mdpi.com/article/10.3390/nano11123431/s1, Figure S1: Surface topography image of (a) the normal FEP film and (b) the polished FEP film, Figure S2: Output performances of the CHR-TENGs. (a) The short-circuit current and (b) transfer charge of the CHR-TENGs with different reflector sizes under the same acoustic wave condition with the acoustic frequency ranging from 40 to 200 Hz, Figure S3: Output performances of the CHR-TENGs. (a) The short-circuit current and (b) transfer charge of the CHR-TENGs with different resonator thicknesses under the same acoustic wave condition with the acoustic frequency ranging from 40 to 250 Hz, Figure S4: Output per-formances of the CHR-TENGs. (a) The short-circuit current and (b) transfer charge of the CHR-TENGs with different coniform angles under the same acoustic wave conditions, with the acoustic frequency ranging from 20 to 200 Hz, Figure S5: Output performances of the CHR-TENGs. (a) The short-circuit current and (b) transfer charge of the CHR-TENGs with varied FEP film sizes under the same acoustic wave conditions, with the acoustic frequency ranging from 20 to 400 Hz. Figure S6. Schematic diagram of the CHR-TENG harvesting ship acoustic energy in a ship engine room. (a) open-circuit voltage and (b) short-circuit current. Figure S7. Power output performance of the CHR-TENG under the acoustic pressure level of 90 dB and acoustic frequency of 140Hz. Figure S8. Test of the stability output performance of the CHR-TENG under acoustic condition of sound pressure level 90dB and frequency 140Hz. (a) single test result and (b) comparison of the three day’s output performance test. Video S1: Demonstration of the CHR-TENG powering a thermometer. Video S2: Demonstration of the CHR-TENG powering 0.5W LEDs. Video S3: Demonstration of CHR-TENG powering 464 LEDs. Table S1: Acoustic levels in various transportation scenarios. Table S2: Power density comparison with previously acoustic energy harvesters.
