# Supplementary material for: A High-Performance Coniform Helmholtz Resonator-Based Triboelectric Nanogenerator for Acoustic Energy Harvesting
Source: Nanomaterials (Basel). 2021 Dec 17;11(12):3431. doi: 10.3390/nano11123431 (PMC8705617; doi:10.3390/nano11123431)
Supplement: Supplementary file 1 [file nanomaterials-11-03431-s001.zip › nanomaterials-1463090-supplementary.pdf]

A High-Performance Coniform Helmholtz Resonator-Based Triboelectric Nanogenerator for Acoustic Energy Harvesting

Haichao Yuan [1,†], Hongyong Yu [1,†], Xiangyu Liu [1], Hongfa Zhao [2], Yiping Zhang [1], Ziyue Xi [1], Qiqi Zhang [1], Ling Liu [1], Yejin Lin [1,*], Xinxiang Pan [1,3,*] and Minyi Xu [1,*]

[1] Dalian Key Lab of Marine Micro/Nano Energy and Self-Powered System, Marine Engineering College Dalian Maritime University, Dalian 116026, China; yuanhc@dlmu.edu.cn (H.Y.); yuhongyong2020@dlmu.edu.cn (H.Y.); simonlxy@dlmu.edu.cn (X.L.); zyp672216686@dlmu.edu.cn (Y.Z.); yyds@dlmu.edu.cn (Z.X.); qiqizhang@dlmu.edu.cn (Q.Z.); pinky@dlmu.edu.com (L.L.); linyj@dlmu.edu.cn (Y.L.)

[2] Tsinghua-Berkeley Shenzhen Institute, Tsinghua Shenzhen International Graduate School, Tsinghua University, Shenzhen 518055, China; zhaohf21@mails.tsinghua.edu.cn

[3] School of Electronics and Information Technology, Guangdong Ocean University, Zhanjiang 524088, China

* Correspondence: linyj@dlmu.edu.cn (Y.L.); panxx@dlmu.edu.cn (X.P.); xuminyi@dlmu.edu.cn (M.X.)

† Haichao Yuan and Hongyong Yu equally contributed to this work.

Supporting Figures

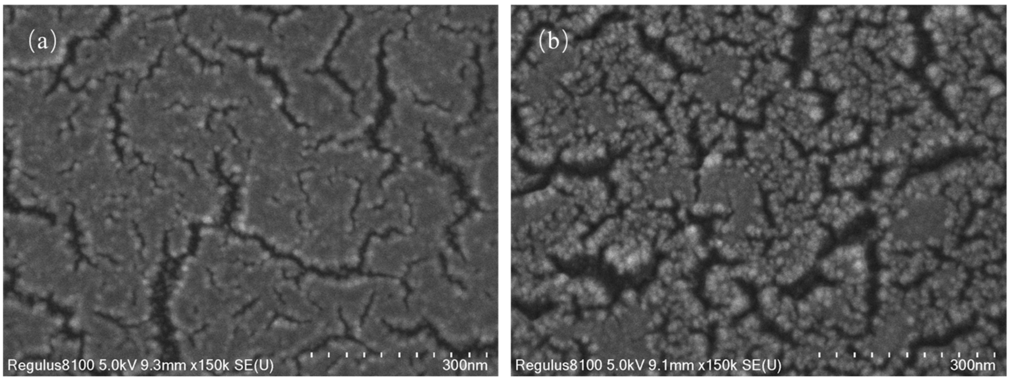

Figure S1. Surface topography image of (**a**) the normal FEP film and (**b**) the polished FEP film.

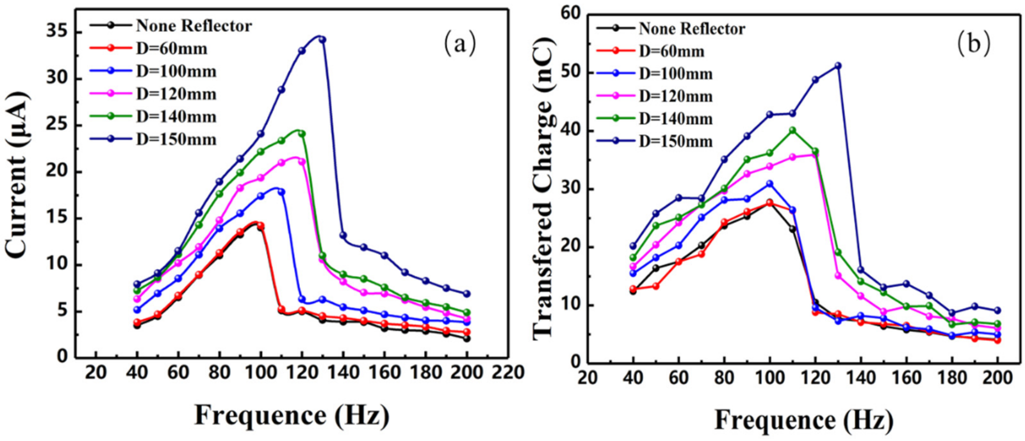

Figure S2. Output performances of the CHR-TENGs. (**a**) The short-circuit current and (**b**) transfer charge of the CHR-TENGs with different reflector sizes under the same acoustic wave condition with the acoustic frequency ranging from 40 to 200 Hz.

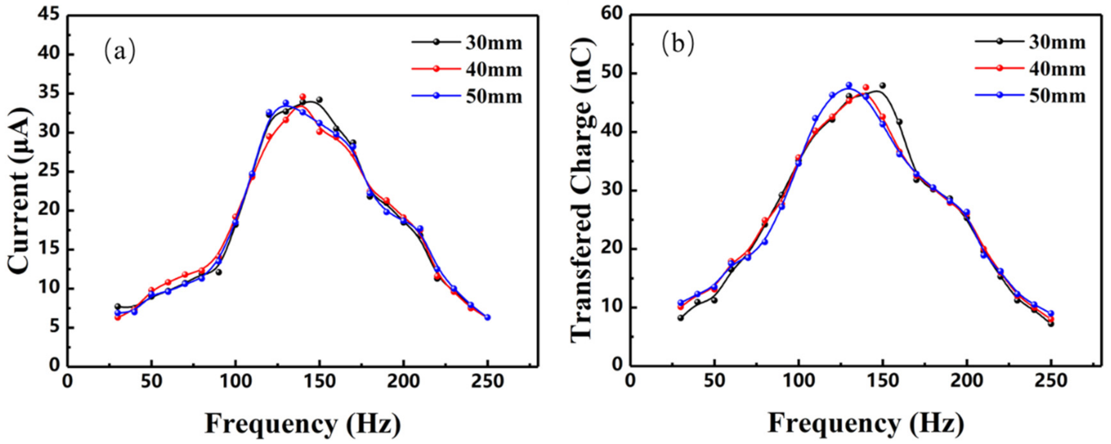

Figure S3. Output performances of the CHR-TENGs. (**a**) The short-circuit current and (**b**) transfer charge of the CHR-TENGs with different resonator thicknesses under the same acoustic wave condition with the acoustic frequency ranging from 40 to 250 Hz.

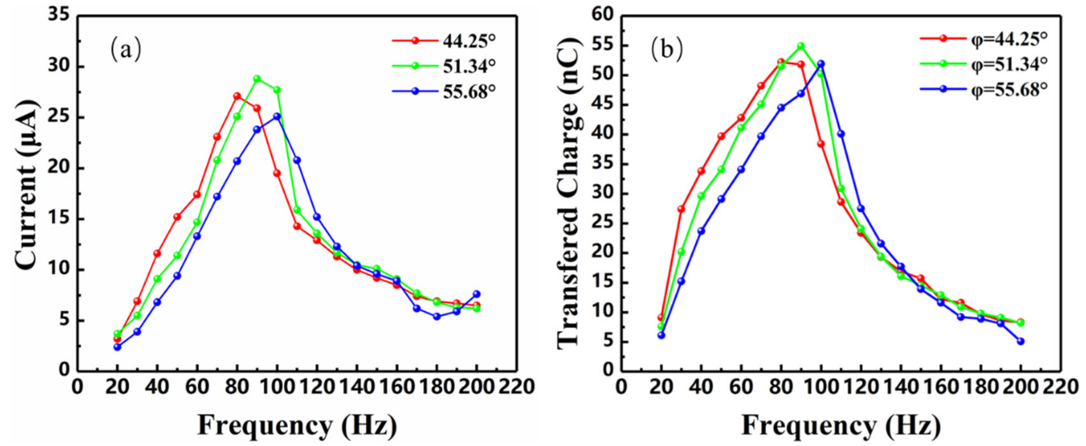

Figure S4. Output performances of the CHR-TENGs. (**a**) The short-circuit current and (**b**) transfer charge of the CHR-TENGs with different coniform angles under the same acoustic wave condition with the acoustic frequency ranging from 20 to 200 Hz.

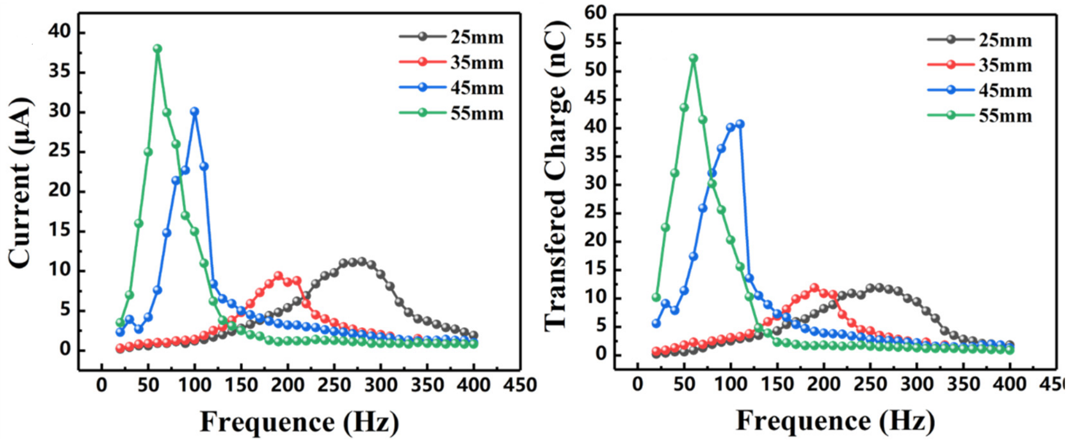

(**a**) (**b**)

Figure S5. Output performances of the CHR-TENGs. (**a**) The short-circuit current and (**b**) transfer charge of the CHR-TENGs with varied FEP film sizes under the same acoustic wave condition with the acoustic frequency ranging from 20 to 400 Hz.

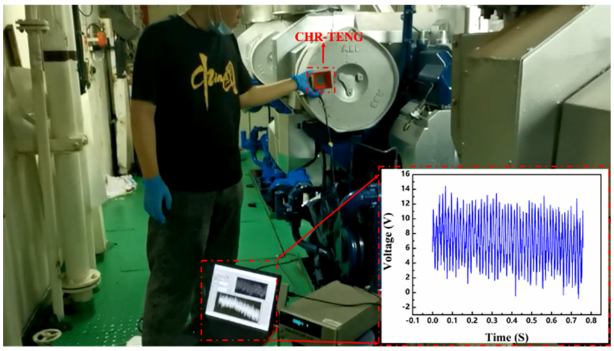

(**a**)

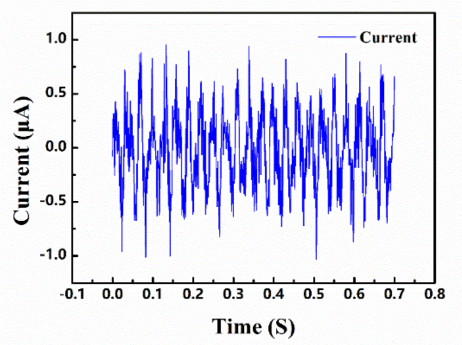

(**b**)

Figure S6. Schematic diagram of the CHR-TENG harvesting ship acoustic energy in a ship engine room. (**a**) open-circuit voltage and (**b**) short-circuit current.

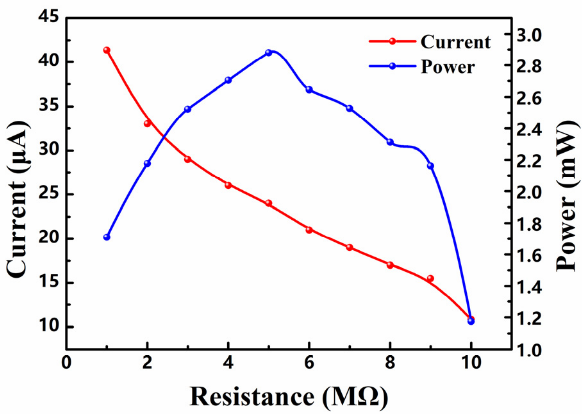

Figure S7. Power output performance of the CHR-TENG under the acoustic pressure level of 90 dB and acoustic frequency of 140Hz.

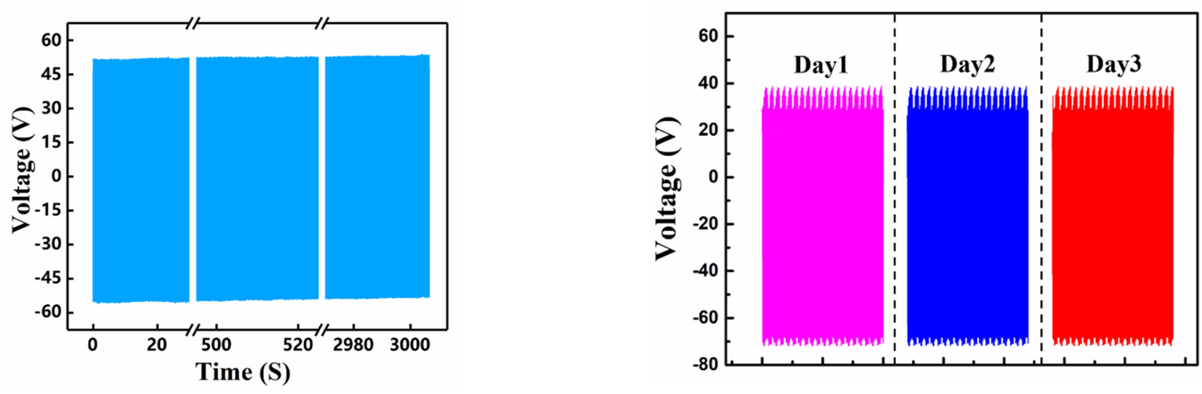

(**a**) (**b**)

Figure S8. Test of the stability output performance of the CHR-TENG under acoustic condition of sound pressure level 90dB and frequency 140Hz. (**a**) single test result and (**b**) comparison of the three day's output performance test.

Simulation conditions of COMSOL Multiphysics:

1. Ideal conditions: It is assumed that the sound pressure from the speaker is the incident sound pressure, regardless of the loss into the device; the pressure behind the membrane is the standard atmospheric pressure.

2. In the simulation, it is assumed that the membrane is rigid, there is no displacement, and only the sound pressure level is concerned.

3. The sound moves in one direction, regardless of sound refraction.

Supporting Tables

Table S1. Acoustic levels in various transportation scenarios.

Acoustic levels	Airport	Ship	Road
Acoustic pressure	normally about 75dB, over 100dB when airplane take-off or landing	over 100dB (when the diesel engine running)	60-90db
Acoustic frequency	＜250Hz	31.5~500Hz	63Hz（corresponding frequency of the maximum noise）

Table S2. Power density comparison with previously acoustic energy harvesters.

No.	Author(s)	Type	Acoustic condition	Electrical output	Dimension (mm)	Ref. No.
1	F. U. Khan and Izhar	Electromagnetic method	144 Hz<br>120 dB	315 mV,<br>1.503 mW	Magnet 1.6×6.35<br>Coil 33×12	[46]
2	S. Qi	Acoustic metamaterial with piezoelectric material	2257.5 Hz<br>100 dB	1.3 V<br>8.8 μW	60×60×5.4	[9]
3	S. N. Cha	Piezoelectric nanowire	100 Hz<br>100 dB	50 mV<br>0.3 μW	10 × 10	[44]
4	B. Li	PZT piezoelectric plates	199 Hz<br>100 dB	1.75 V<br>0.65 mW	40×20×0.48	[47]
5	A. Yang	PZT plate with Sonic Crystal and Helmholtz resonator	5.545 KHz<br>110 dB	3.89 V<br>429 μW	32 ×12	[13]
6	X. Fan	TENG with PTFE	320 Hz<br>117 dB	About 79 V<br>About 8.5 mA	Not specified	[34]
7	J. Yang	TENG with PTFE	240 Hz	60.5 V	Radius 65	[45]

			110 dB	15.1 μA		
8	N. Cui	TENG with PVDF	175 Hz<br>114 dB	90 V<br>0.45 mA	100×100	[40]
9	J. Liu	TENG with PVDF	200 Hz<br>105 dB	232 V<br>More than 1<br>mA	1 and 5 plates<br>95×95	[48]
10	F. Chen	TENG with PVDF	170 Hz<br>115 dB	400 V<br>175 μA	Radius 80	[32]
11	H.Zhao	TENG with FEP	80 Hz<br>89.1 dB	140 V<br>60 μA	45× 45 ×0.05	[33]
12	M.Yuan	TENG with FEP	88Hz<br>100dB	4.33mW	Radius 80	[31]
13	Z.Wang	TENG with FEP	170Hz<br>110dB	500V<br>124μA	Radius 80	[41]
14	Present	TENG with FEP	140Hz<br>90dB	167V<br>48μA	Radius 45	
